# Multipoint Temperature Monitoring of Microwave Thermal Ablation in Bones through Fiber Bragg Grating Sensor Arrays †

**DOI:** 10.3390/s20113200

**Published:** 2020-06-04

**Authors:** Elena De Vita, Martina Zaltieri, Francesca De Tommasi, Carlo Massaroni, Eliodoro Faiella, Bruno Beomonte Zobel, Agostino Iadicicco, Emiliano Schena, Rosario Francesco Grasso, Stefania Campopiano

**Affiliations:** 1 Department of Engineering, University of Naples “Parthenope”, Centro Direzionale Isola C4, 80143 Naples, Italy; elena.devita@uniparthenope.it (E.D.V.); iadicicco@uniparthenope.it (A.I.); 2 Department of Engineering, Università Campus Bio-Medico di Roma, Via Alvaro del Portillo, 00128 Rome, Italy; m.zaltieri@unicampus.it (M.Z.); francesca.detommasi95@gmail.com (F.D.T.); c.massaroni@unicampus.it (C.M.); 3 School of Medicine, Università Campus Bio-Medico di Roma, Via Alvaro del Portillo, 00128 Rome, Italy; e.faiella@unicampus.it (E.F.); b.zobel@unicampus.it (B.B.Z.); r.grasso@unicampus.it (R.F.G.)

**Keywords:** temperature measurements, fiber bragg grating sensors, fiber optic sensors, thermal ablation of cancer, microwave thermal ablation, microwave ablation of bone

## Abstract

Bones are a frequent site of metastases that cause intolerable cancer-related pain in 90% of patients, making their quality of life poor. In this scenario, being able to treat bone oncology patients by means of minimally invasive techniques can be crucial to avoid surgery-related risks and decrease hospitalization times. The use of microwave ablation (MWA) is gaining broad clinical acceptance to treat bone tumors. It is worth investigating temperature variations in bone tissue undergoing MWA because the clinical outcomes can be inferred from this parameter. Several feasibility studies have been performed, but an experimental analysis of the temperature trends reached into the bone during the MWA has not yet been assessed. In this work, a multi-point temperature study along the bone structure during such treatment is presented. The study has been carried out on ex vivo bovine femur and tibia, subjected to MWA. An overall of 40 measurement points covering a large sensing area was obtained for each configuration. Temperature monitoring was performed by using 40 fiber Bragg grating (FBGs) sensors (four arrays each housing 10 FBGs), inserted into the bones at specific distances to the microwave antenna. As result, the ability of this experimental multi-point monitoring approach in tracking temperature variations within bone tissue during MWA treatments was shown. This study lays the foundations for the design of a novel approach to study the effects of MWA on bone tumors. As consequence, the MWA treatment settings could be optimized in order to maximize the treatment effects of such a promising clinical application, but also customized for the specific tumor and patient.

## 1. Introduction

During the last decades, the increase of population life expectancy combined with unhealthy lifestyles and poor food habits led to a greater occurrence of tumors cases, especially in developed countries [1]. In many instances, patients who need cancer treatments are aged or have complicated medical histories caused by pre-existing conditions. In this scenario, treating cancer patients using minimally invasive techniques (MITs) to minimize surgery-related risks and decrease hospitalization times is highly preferable, since 20% of the patients undergoing radiotherapy, the gold standard in this kind of treatment, are unresponsive in terms of pain control. In this respect, MITs are gaining momentum and wide clinical acceptance for the treatment of several kinds of tumors (i.e., liver, lung, kidney and pancreas) located in different body districts [2]. Microwave ablation (MWA), laser ablation (LA), and radiofrequency ablation (RFA) are MITs that induce a necrosis of the target tissue cells through hyperthermia. A certain amount of energy is released into the target organ by one or multiple needlelike applicators percutaneously inserted. A considerable temperature increment is caused by the interplay between the delivered energy and the biological tissue. At certain values (typically above 50 °C [3]), such temperature rises are cytotoxic and lead to irreversible cellular damage. For a good outcome of the procedure, it is also necessary to induce necrosis in an additional margin of healthy tissue surrounding the tumorigenic area. Such additional margin should be sufficiently thick to ensure successful treatment results, but thin enough to minimally damage the surrounding healthy structures (ranges approximately from 5 mm to 10 mm [3,4]).

Thermal treatments of bone tumors and metastases is a recent clinical field which is gaining great interest due to its promising medical outcomes [5,6,7]. In fact, 90% of bone cancer patients suffer from intolerable cancer-related pain that can be relieved by such treatments, leading to an improvement in the quality of life [8]. In the context of orthopedic microsurgery, MWA practices are becoming ever more popular [8,9,10].

As in all thermoablation procedures, in MWA treatments temperature propagation modality, as well as the treatment time, the applicator geometry and the mechanism of energy delivery, affect the shape and the volume of the necrotic tissue [11]. Therefore, being able to measure the temperature trend over time during the whole treatment can be useful to investigate the effect of the treatment in terms of lesion size and to optimize the clinical results. In addition, temperature knowledge may be beneficial to correct the results of hyperthermia treatment planning tools adopted to make the treatment patient-specific [12]. Finally, temperature monitoring can be crucial to assess the performance of new heating devices and to guide their design. These considerations are particularly relevant for new or recent applications such as MWA on bones. Although several thermometric techniques have been used to investigate temperature increments during thermal procedures on several organs [13,14], to date little experimental data on the temperature distribution on bones undergoing MWA have been reported in literature [15,16]. In both these two studies, the experiments employed a thermocouple, so a single-point temperature measurement was performed. Since, in this framework it is worth investigating a three-dimensional temperature distribution to gain a deeper knowledge of the phenomenon, multi-point temperature measurements are recommended. Among several techniques, fiber Bragg grating sensors (FBGs) allow performing highly resolved and multi-point temperature measurements [17,18,19].

In the present study, the assessment of FBGs feasibility for executing multi-point temperature measurements into bones undergoing MWA procedures is reported. Experiments were performed on ex vivo bovine bones by monitoring temperature trends at 40 measurement sites. 

## 2. Materials and Methods

In this section, the experimental setup used to perform multi-point temperature measurements during MWA on ex vivo bovine femur and tibia is described.

### 2.1. Microwave Ablation of Bovine Bone: Principle, Experimental Setup and Settings

**Figure 1:**
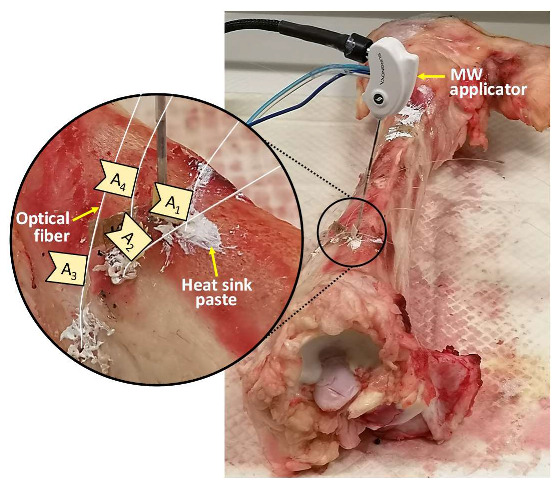
Photo of the experimental setup used during the ablation of the bovine tibia; for the sake of clarity, the insertion site of microwave (MW) applicator and optical fibers is zoomed in on.

Ex vivo bovine femur and tibia were subjected to MWA treatment in the trochanter and in the meta-diaphyseal part, respectively. An M150E Generator (Microwave Ablation System, Surgnova Healthcare Technologies, Zhejiang, China) and a 150 mm long antenna (SS-MWA-1531C, Microwave Ablation Electrode Kits, Surgnova Healthcare Technologies, Zhejiang, China) emitting at 2.45 GHz frequency were used. The MW antenna was inserted into a hole created in the middle of the bone structure with a bone biopsy stiff cannula 13 G calipered, the same adopted during interventional bone procedure. The tip of the applicator was positioned at a depth of about 40 mm with respect to the bone surface. Then, four optical fibers, each embedding an array of 10 FBGs, were positioned in four holes placed at different distances from the MW antenna. For this purpose, five access points were created on the surface of the bone tissue by means of a diamond-tipped needle at first, then the holes for the insertion of antenna and optical fiber sensors were excavated by using a driller. The insertion of the optical fibers in the holes of the bone tissue involved the use of a heat sink paste in order to increase the thermal conductivity between fiber and bone tissue, as shown in Figure 1. As detailed in the following, the coordinate system was chosen so that the origin was set on the antenna tip and the *z*-axis matched with the antenna. 

The experiments were performed at room temperature (i.e., about 17 °C measured in proximity of the bone surface right before starting the procedure) setting a power of 75 W (which corresponds to a power delivered by the antenna of approximately 52 W) for 8 min (i.e., 480 s) of treatment time. 

### 2.2. Temperature Monitoring during Microwave Ablation: Principle, Experimental Setup and Settings

Four fiber optics, each housing an array of 10 FBG sensors, were inserted into the bone thereby obtaining 40 measurement sites. Each sensor (AtGrating Technologies, Shenzhen, China) was characterized by a reflectivity value ranging from 60% to 65%. Gratings were 1 mm long and equally spaced 2 mm from each other for a total sensitive length of 28 mm. 

Once illuminated by a broadband spectrum source, an FBG sensor embedded into the fiber core transmits the light, except for a narrow spectrum which is reflected backward. Such spectrum is centered around the Bragg wavelength (*λ_B_*) [20], different for each sensor, which can be expressed as follows:

(1) $$\lambda_{B}=2\cdot \eta_{eff}\cdot \Lambda$$

where *Λ* is the grating period and *η_eff_* is the core effective refractive index. 

As the shift detected in *λ_B_* (Δ*λ_B_*/*λ_B_*) is linearly related to temperature variations through the thermal sensitivity, whose value is 6.5·10^−6^ °C^−1^, the sensing element is able to detect temperature variation with a resolution of 0.1 °C.

To analyze the FBG arrays, an optical spectrum interrogator (Optical Sensing Interrogator, si255 based on Hyperion Platform, Micron Optics, Atlanta, GA, USA), capable of discriminating all the 40 λ_B_ corresponding to each sensor, was used. The temperature trend related to the 40 measurement sites was so monitored during the whole MWA process (i.e., 480 s) and the cooling phase (about 300 s after turning off the microwave antenna). All the data were collected at a sampling frequency of 100 Hz and then analyzed in MATLAB^®^ (Mathworks, Natick, MA, USA) environment. The temperature increment was calculated with respect to the temperature value recorded right before starting the MWA procedure.

### 2.3. Sensors and Antenna Positioning during Microwave Ablation

**Figure 2:**
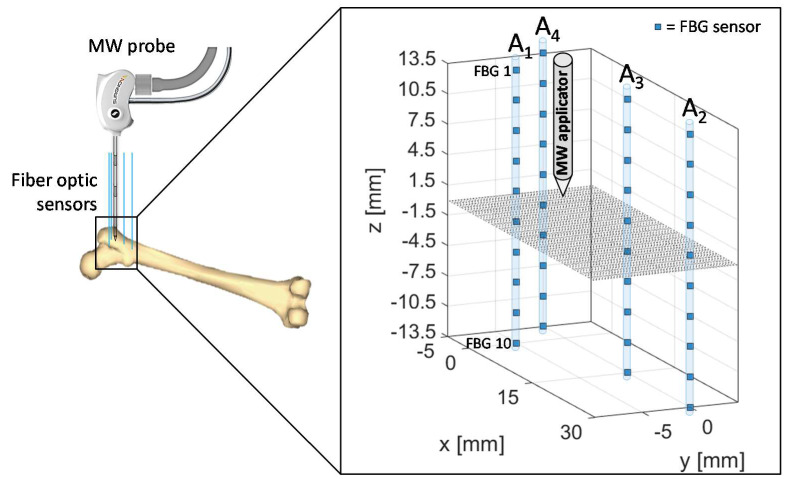
Schematic of fiber Bragg grating (FBGs) and antenna positioning during the microwave ablation (MWA) procedure in the trochanteric tissue of a bovine femur. The antenna tip represents the origin of the coordinate system.

For the experimental test on bovine femur, the MW antenna and 40 FBGs were inserted in the tissue as shown in Figure 2, in correspondence of the trochanter. The 10 FBGs of each array are denoted by a progressive number starting from the top of *z*-axis. The tip of the antenna was positioned at the same depth of the center of the arrays, namely between the fifth and the sixth FBG of each array, at a depth of about 40 mm with respect to the bone surface.

Therefore, the relative positioning of the antenna and sensors allowed to cover a measurement area which extends for 14 mm along the distal part of the antenna and for 14 mm below the applicator tip along the *z*-axis, with 4 arrays of sensors distributed along *x*- and *y*-axes.

Array coordinates in the *x*-*y* plane are the following:

- A_1x_ = 0 mm, A_1y_ = −5 mm
- A_2x_ = 30 mm, A_2y_ = 0 mm
- A_3x_ = 15 mm, A_3y_ = 0 mm
- A_4x_ = −5 mm, A_4y_ = 0 mm

The proposed setup and sensors configuration allowed us to estimate temperature trends reached by the trochanteric tissue at different depths and distances from the applicator. 

**Figure 3:**
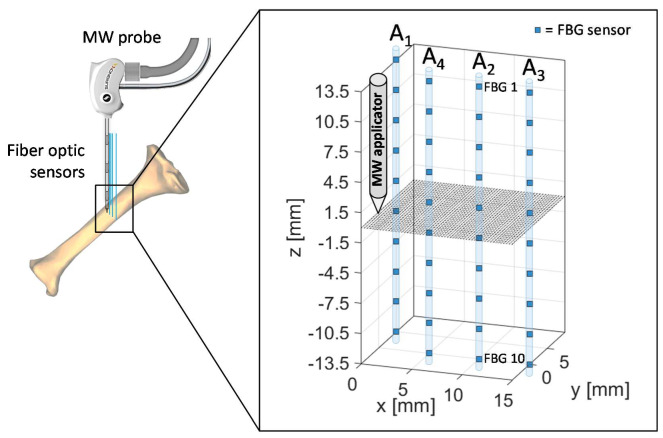
Schematic of FBGs and antenna positioning during the MWA procedure in the meta-diaphyseal part of a bovine tibia. Antenna tip represents the origin of the coordinate system.

The spatial configuration of sensors and MW applicator applied during the experimental test performed on ex vivo bovine tibia is outlined in Figure 3. The same four arrays of FBG were employed, for a total 40 measurement sites, and antenna tip, corresponding at the origin of the coordinate system, was positioned at the same depth of the center of the arrays also in this case, at a depth of about 40 mm with respect to the bone surface. 

Array coordinates in the *x*-*y* plane are the following:

- A_1x_ = 0 mm, A_1y_ = 5 mm
- A_2x_ = 10 mm, A_2y_ = 0 mm
- A_3x_ = 15 mm, A_3y_ = 0 mm
- A_4x_ = 5 mm, A_4y_ = 0 mm

The proposed setup and sensors configuration allowed us to estimate temperature trends reached by the tibia at different depths and distances from the applicator.

## 3. Results

In this section, the experimental results are reported and discussed. Two different bovine bones (i.e., femur and tibia) undergoing MWA were investigated to measure and map the temperature profiles reached within the tissue.

### 3.1. MWA on Ex Vivo Bovine Femur

**Figure 4:**
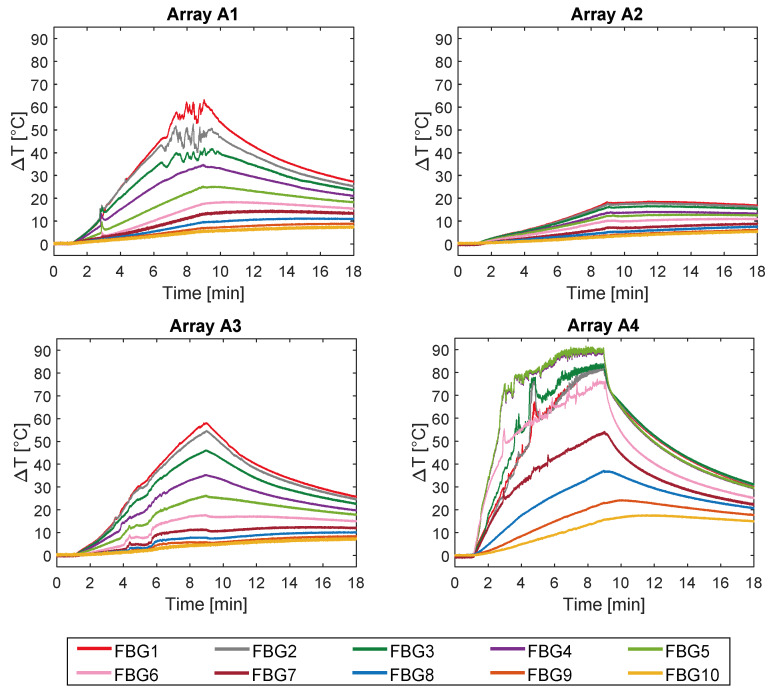
Temperature variations collected during the MW discharge and the cooling phase. The ten measurements recorded by the ten FBGs embedded in array A_1_ and A_4_ (both 5 mm far from the applicator), in array A_2_ (30 mm far from the applicator), and in A_3_ (15 mm far from the applicator) are shown.

The temperature profiles collected by the 40 FBGs during the experiments on bovine femur are shown in Figure 4.

**Figure 5:**
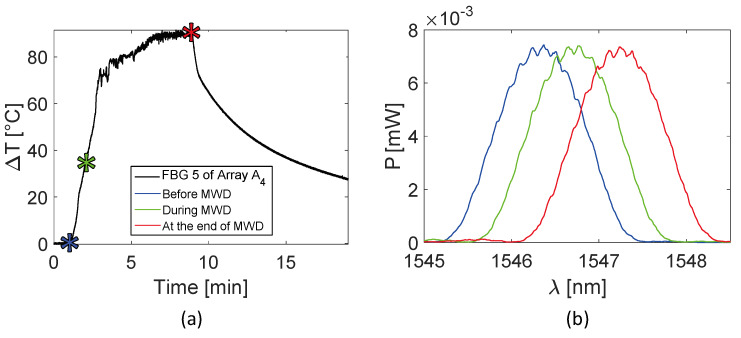
Focus on the fifth FBG belonging to array A_4_, the sensor which recorded the maximum temperature value during the MWA treatment: (**a**) temperature variation versus time; (**b**) optical reflected spectra before, during and at the end of the MW discharge (MWD) in the three different instants of time marked in (**a**), plotted in blue, green and red respectively.

Trends of temperature variation recorded by the FBGs of the arrays close to the antenna (A_1_ and A_4_, 5 mm distant from the antenna, see Figure 2) show a significant increase which started shortly after the MW generator was turned on and lasted during the whole treatment (480 s). Along the sensitive area, 28 mm long of arrays A_1_ and A_4_, the maximum temperature increments recorded during the MW discharge by the 10 FBGs of each array ranged from 6.5 to 63.2 °C and from 17 to 91.4 °C, respectively, depending on the distance from the antenna. Results also show that some sensors nominally placed at the same distance from the antenna experienced different temperature. Moving away from the applicator, temperature increments significantly decrease: the maximum variation of temperature recorded by array A_3_, located 15 mm far from antenna (see Figure 2), was 58.1 °C, whereas for array A_2_, placed 30 mm far from the antenna, the maximum temperature value was only 18.5 °C. As the antenna was turned off, the cooling phase of the tissue is evident, especially for arrays A_1_, A_3_, and A_4_, since in the proximity of the applicator the temperature gradient is significant. Among all the sensors, the fifth FBG of A_4_ recorded the highest temperature value; its temperature profile has been reported in Figure 5a and three instants of time have been chosen for comparison: right before, during and at the end of the MW discharge, denoted by blue, green and red stars (Figure 5a), respectively. In Figure 5b, the respective profiles of the optical reflected spectrum, plotted in the corresponding color, are shown, aiming at observing the *λ_B_* shift due to the temperature variation, with the preservation of the spectrum shape over time.

**Figure 6:**
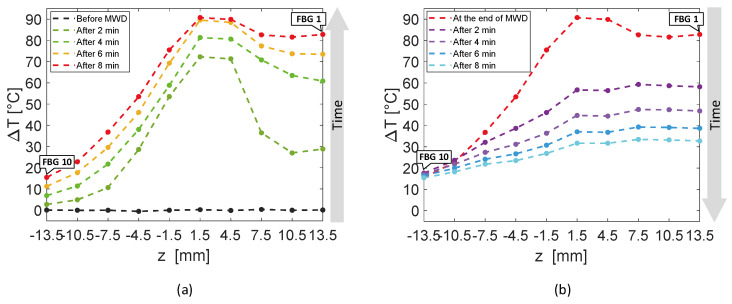
Temperature variations recorded by the ten FBGs of array A_4_ versus distance, along the axis of the array, parallel to the *z*-axis: (**a**) rising temperature profiles recorded every 2 min, starting before the antenna activation to the end of the MW discharge (MWD), which happened 8 min later; (**b**) decreasing temperature profiles starting from the antenna shutdown and recorded 2 min, 4 min, 6 min and 8 min later.

Figure 6 reports an analysis of the temperature values reached by the sensors of the entire array A_4_ with respect to *z*-axis, rather than time. The experimental temperature values recorded by each FBG are represented by dots, whereas dashed lines in the respective color are the linear interpolation of the data. In Figure 6a, rising temperature profiles are shown every 2 min, starting from the black curve, which corresponds to the spatial temperature trend of array A_4_ before the MW discharge, to red profile, recorded after 8 min, at the end of the discharge. Similarly, Figure 6b shows the decreasing spatial profiles of temperature for each sensor belonging to A_4_, starting from the end of the MW discharge and plotting temperature trends every 2 min until 8 min after the MW generator was turned off.

The first five FBGs of A_4_ recorded the highest temperatures since they were the sensors closest to the antenna (see Figure 2). Indeed, Figure 6a shows their progressive rising until reaching the maximum values, i.e., FBG 1 moved up to 82.7 °C, while FBG 10 reached only 15.4 °C at the end of the discharge. As mentioned previously, the maximum temperature variation was achieved by the fifth FBG of the array, located nearly at the same depth of antenna tip.

**Figure 7:**
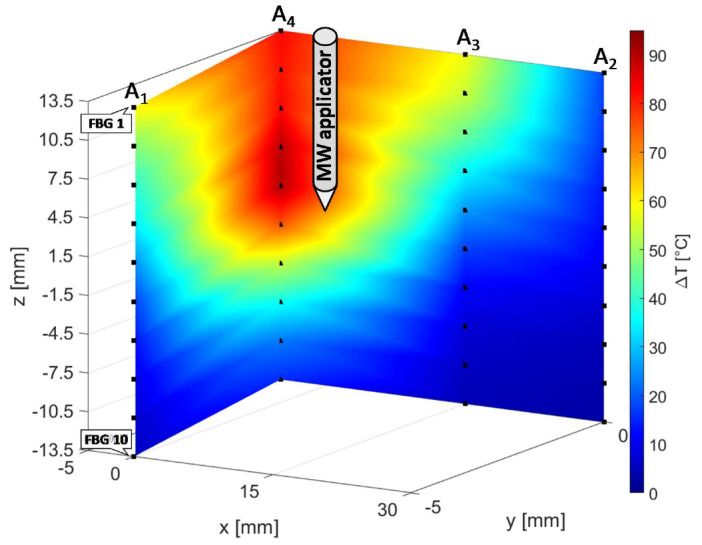
Temperature map in y=0 and x+y=−5 planes; black dots indicate experimental values (not to scale).

Due to use of FBG sensors, multi-point temperature monitoring approach allowed the estimation of the volumetric temperature distribution inside the thermal lesion and the visualization of 3D temperature maps during MWA. Figure 7 reports the temperature map obtained at the end of the MW discharge, when the sensors reached their maximum temperature values, in y=0 plane, to which arrays A_4_, A_3_, and A_2_ belong, and in x+y=−5 plane, in which arrays A_4_ and A_1_ are located. For the sake of clarity, the axes are not reported to scale. Temperature is reported by a colormap versus spatial axes and map was achieved with a linear interpolation of the experimental data points. Temperature profile of array A_4_ along its axis is the same as the spatial temperature distribution plotted in Figure 6a (red profile): temperature decays downwards the array axis, with a maximum (91.4 °C) in correspondence of the fifth FBG. A decreasing trend along the array axis can be observed also for A_1_ and A_3_, which reached the maximum temperature value in correspondence of their first FBG (59.7 °C and 57.9 °C, respectively), and for A_2_, to a small extent as expected since it was 30 mm far from antenna. The overall result pointed out by the three-dimensional mapping is a temperature distribution with a maximum spatial extension of about 20 mm and with maximum located at the correspondent depth of antenna distal part.

### 3.2. MWA on Ex Vivo Bovine Tibia 

**Figure 8:**
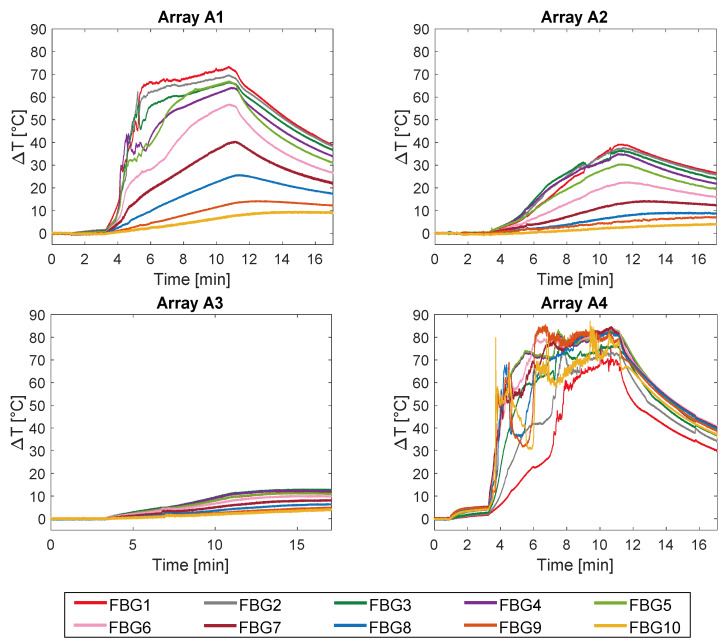
Temperature variations collected during the MW discharge and the cooling phase. The ten measurements recorded by the ten FBGs embedded in array A_1_ and A_4_ (both 5 mm far from the applicator), in array A_2_ (10 mm far from the applicator), and in A_3_ (15 mm far from the applicator) are shown.

The experiments performed on bovine tibia showed a maximum temperature variation measured by the fifth FBG of A_4_ of 83.3 °C at the end of the MWA procedure (Figure 8).

**Figure 9:**
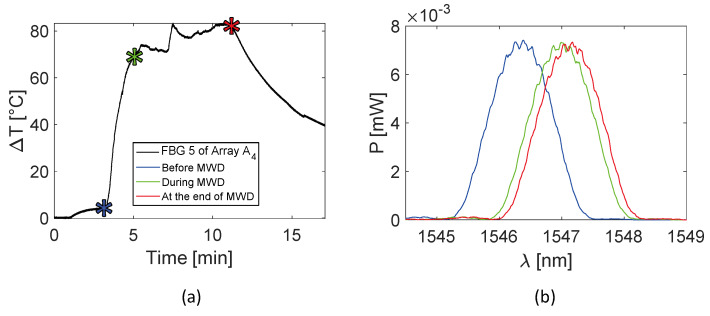
Focus on the fifth FBG of array A_4_, the sensor which recorded the maximum temperature value during the MWA treatment: (**a**) temperature variation versus time; (**b**) optical reflected spectra before, during and at the end of the MW discharge (MWD), in the three different instants of time marked in (**a**), plotted in green, blue and red respectively.

In this case, from the eighth to the tenth FBG of Array A4, the temperature variation profiles are less smooth in comparison to those reported in Figure 4. It is reasonable to believe that in this case it is due to the proximity to the bone marrow, which melts at high temperature, dripping from the hole in the bone tissue. The temperature profile of the fifth FBG of the array A_4_ is represented in Figure 9a. Three instants of time, before, during and at the end of the MW discharge, have been chosen and denoted by blue, green and red stars (see Figure 9a). Figure 9b shows the respective profiles of the optical reflected spectrum, plotted in the corresponding color, pointing out the *λ_B_* shift as a result of temperature variation and the shape preservation of the spectrum over time.

**Figure 10:**
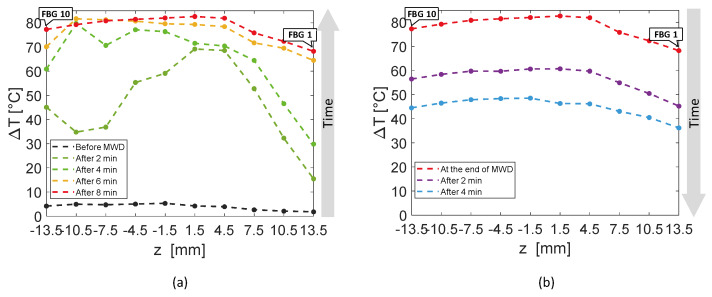
Temperature variations recorded by the ten FBGs of array A_4_ versus space along the axis of the array, parallel to the *z*-axis: (**a**) rising temperature profiles recorded every 2 min, starting before the antenna activation to the end of the MW discharge (MWD), which happened 8 min later; (**b**) decreasing temperature profiles starting from the antenna shutdown and recorded 2 min and 4 min later.

Figure 10 shows the spatial temperature profiles along *z*-axis obtained considering the entire array A_4_. Dots represent the experimental data, linearly interpolated by dashed lines in the corresponding color. The black temperature profile plotted in Figure 10a is almost flat, since it was recorded right before the MW discharge; then, Figure 10a continue with temperature profiles versus array axis recorded every 2 min until reaching the red curve at the end of MW discharge. In Figure 10b, decreasing spatial profiles of temperature measured by A_4_ are shown every 2 min starting from the end of MW discharge. 

Two minutes after thermal treatment beginning (Figure 10a), a temperature peak was achieved in correspondence of the fourth and the fifth FBG of array A_4_, which reached 68.6 and 69.2 °C, respectively, whereas the first FBG measured 15.5 °C only. Then, temperature profiles extended along the whole array (28 mm long), reaching temperature values from 68.3 °C (measured by the first FBG) to 83.3 °C (reached by the fifth FBG).

**Figure 11:**
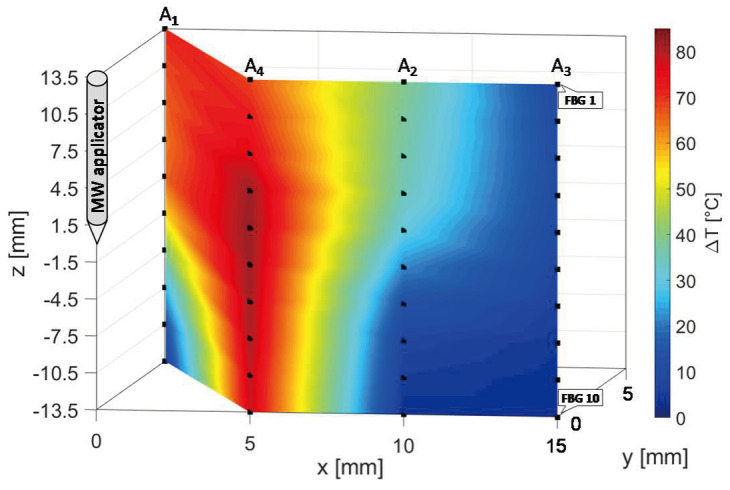
Temperature map in y=0 and x+y=5 planes; black dots indicate experimental values (not to scale).

In Figure 11, the three-dimensional temperature map obtained at the end of the MW discharge is reported not to scale for the sake of clarity. Temperature is reported by a colormap versus spatial axes and map was achieved with a linear interpolation of the experimental data points in y=0 plane, containing arrays A_2_, A_3_ and A_4_, and in x+y=5 plane, containing arrays A_1_ and A_4_.

**Figure 12:**
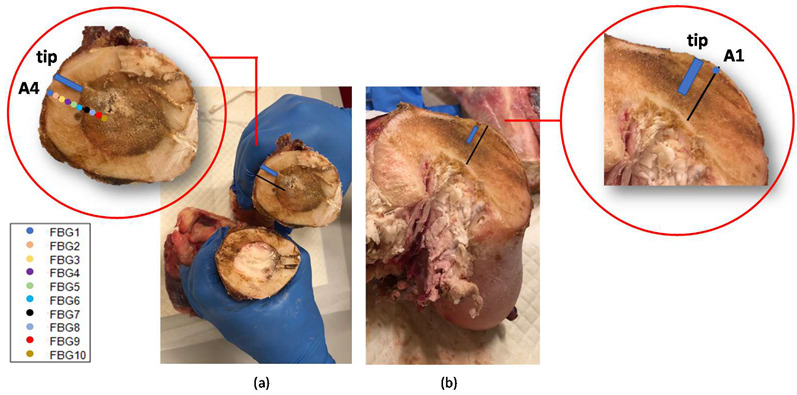
Bones dissections after the MWA treatments. The blue rectangle indicates the antenna tip position in the bone section and the colored markers in the insets indicate the FBGs located in same bone section measuring temperatures higher than 60 °C. In (**a**), a diaphyseal section: in correspondence of the MW antenna site (where the bone marrow became brown as was damaged by the heat) and far from the antenna (where the bone marrow shows its natural color). In (**b**), the section in correspondence with the trochanter.

At the end of the MW treatment, all the sensors of array A_4_ measured the highest temperatures in the map, followed by A_1_, which reached a decreasing spatial profile of temperature variation along its axis, from 71 °C (FBG 1) to 8.1 °C (FBG 10). The maximum spatial extension of the thermo-ablated region was of about 10 mm, since starting from a distance of 10 mm from the antenna the three-dimensional map in Figure 12 shows a cooling phenomenon. In fact, at 10 mm and at 15 mm from the MW applicator lower temperature variations from 39.0 to 2.8 °C and from 10.0 to 2.7 °C were measured by arrays A_2_ and A_3_, respectively.

The bone dissection performed at the end of the treatment shows the effect on the bones of the MWA (Figure 12). The bone marrow damaged by the heat confirms the high temperature values reached during the MWA. Colored markers indicate the FBGs located in such bone section which measured above 60 °C, whereas the blue rectangle indicates antenna tip.

## 4. Discussion and Conclusions

MWA is a MIT that is becoming widely used in bone cancer therapy routines [8,9,10]. Treatment time and temperature propagation modality are two of the several factors that mainly influence the size of the necrotic volume and in turn the success of the treatment. As consequence, to investigate the effects of MWA on the target tissues and understand how the treatment settings (i.e., power and treatment time) influence such effects, it is necessary to know the temperature map of the tissue undergoing the MWA. FBGs are considered to be suitable for this application as they provide highly resolved and multi-point temperature measurements [17,18,19]. Despite FBGs’ feasibility for this application having been assessed on soft tissues (e.g., kidney, liver, pancreas) undergoing thermoablation procedures [19,21,22,23,24,25], literature on bones is lacking.

In the present study the feasibility of FBGs to execute multi-point temperature measurements with high spatial resolution has been assessed on bones (i.e., ex vivo bovine femur and tibia) undergoing MWA. A total of four FBGs arrays for a total of 40 measurement points were positioned around the antenna site at different distances (see Figure 1). The maximum temperature values (i.e., 63.2 and 91.4 °C) were recorded approximately at the end of the discharge (at about 480 s of treatment) by the arrays which were closer to the antenna (A1 and A4, respectively), as shown in Figure 4 and Figure 8. Results show that sensors placed at the same distance from the applicator sometimes experienced different temperature increments. This result can be caused by either inhomogeneities of the bone tissue or small errors in the positioning of the sensors. Regarding the positioning, it is worth noting that tiny distance discrepancies can cause significant temperature differences due to the high thermal gradient close to the applicator. For both femur and tibia, the temperature profile of the FBG that reported the highest values (the fifth FBG belonging to A_4_ in both cases) is presented (see Figure 5a and Figure 9a and three instants of times (before MW discharge, during MW discharge and at the end of MW discharge) have been compared by plotting the related spectrum. The Δ*λ_B_* caused by temperature increments is clearly visible, as well as the preservation of the spectrum shape over time. Moreover, the temperature variations recorded by the 10 FBGs embedded in A_4_ are showed (Figure 6 and Figure 10) displaying the temperature peaks reached by each sensor over time. In Figure 7 and Figure 11, the temperature maps obtained at the end of the MWA using a linear interpolation between the experimental data points are reported for the femur and tibia bones, respectively. As shown in Figure 7 and Figure 11, the bone tissue areas subjected to temperatures greater than 50–60 °C [3] are wide, as can also be verified in Figure 12, and the use of FBGs permits to map the temperature in such large areas. Moreover, the temperature map shape obtained for the tibia bone is compatible with the shape of the necrotic tissue assumed in the antenna user manual [26] for the configured treatment settings. As can be observed in Figure 7, the highest temperatures are reached in the areas close to the active part of the emitting antenna and the isothermal curves present an oval shape. On the contrary, for the tibia 3D temperature map (Figure 11) a different shape is obtained since the highest temperatures are recorded both close to the active part of the antenna and in the area immediately below, along the axis of the antenna. Such a behavior can be explained as during the entire treatment a copious flow of liquid (i.e., liquefied marrow due to high temperatures) was observed to come out from the lower part of the bone.

The main limitations of the study are the small number of tests and that the tests were performed in ex vivo healthy bones, but it is novel in terms of working methodology and application.

To date, the temperature trend studies on bones undergoing thermoablation procedures are few and mostly use thermocouples with a single measurement point [15,27]. In these studies, thermocouples were used to avoid serious complications caused by uncontrolled heat expansion in bone tumors closely located to vital structures, such as spinal cord, nerve roots and pericardium. Although successful in the purpose, such an approach was not able to guarantee temperature monitoring along the entire structures. In some cases, as a consequence, adding several thermocouples was necessary. Otherwise, in this work we used four FBGs array for a total of 40 measurements point. This approach is particularly useful to monitor wide bone districts where severe temperature variations could damage the surrounding structures. In fact, the use of FBG technology can indicate in real-time if, in a certain bone area, temperatures exceed specific thresholds, so permitting the operators to suspend the treatment avoiding undesirable damages. Furthermore, the construction of three-dimensional temperature maps with high spatial resolution can enhance the knowledge of the heat diffusion mechanisms into bone tissues. Indeed, thermal 3D maps constitute solid experimental evidence to which to refer for the development of increasingly sophisticated heat diffusion models for thermal ablation procedures. Therefore, this multipoint temperature analysis can also help to assess new heating tools and to compare their effects with consolidated tools. It is also a good starting point to deeply understand how and how far the procedure settings, in terms of power delivered and treatment time, affect the size of the necrotic area.

Future works will be focused on the analysis of the influence of the MWA settings on the size of the necrotic bone tissue, increasing both the number of experiments and the types of bones.
